# Suggested risk factor for oral lichen planus arising after mRNA COVID‐19 vaccination

**DOI:** 10.1002/ccr3.6126

**Published:** 2022-07-22

**Authors:** Massoumeh Zargaran

**Affiliations:** ^1^ Department of Oral and Maxillofacial Pathology, Faculty of Dentistry Kurdistan University of Medical Sciences Sanandaj Iran

## Abstract

A single dose of BNT162b2 vaccine is suggested for COVID‐19‐infected individuals to reduce side effects due to release of inflammatory mediators such as oral lichen planus (OLP).

Dear Editor,

Several vaccines with favorable safety and efficacy profiles administrate against SARS‐CoV‐2 infection. Meanwhile, more knowledge on their possible side effects is surfacing in progress of the vaccination campaign.^1^

BNT162b2 (Pfizer/BioNTech) is one of the mRNA vaccines currently used.^1^

Kaomongkolgit and Sawangarun^2^ reported a case of oral lichen planus (OLP) after receiving the second dose of BNT162b2 vaccine in a person with no past history of OLP. The mechanism responsible for this event is not fully known although a proposed explanation is that BNT162b2 vaccine induces upregulation of Th1 response. This may play a relevant role in lichen planus (LP) activation through an increase in the levels of IL‐2, IFN‐γ, and TNF‐α as common inflammatory cytokines directly involved in the pathogenesis of LP.^3^, ^4^ The exact underlying pathomechanisms need more investigations as it seems some conditions may play a role in enhancing risk of OLP development in vaccine recipients as follow:

After the first BNT162b2 dose of vaccine administration, changes occur in the level of inflammatory cytokines such as IFN‐γ that is positively correlated with the generation rate of anti‐Spike‐RBD antibodies/antibody titers. On the contrary, level of antibody titers is influenced by previous infection (seropositive) or non‐infection (seronegative) status of COVID‐19 before the prime injection in the vaccine recipients.^5^

The first dose of BNT162b2 vaccine increases anti‐spike IgG titers in seropositive more than 140 times of that of peak pre‐vaccine levels. It also significantly increases the antibody titers and strengths of T‐cell responses in seropositive individuals more than seronegative ones.^6^, ^7^

After the second injection of the vaccine, cytokine changes become broader and greater in these seropositive individuals (suggesting stimulation of anamnestic responses). Whereas more obvious role of cytokines induces TNF‐α and IL‐6.^5^

Hence, a single dose of BNT162b2 vaccine is recommended for COVID‐19‐infected individuals. It might be sufficient to induce an effective response^5^, ^6^ as it reduces side effects due to the release of inflammatory mediators.

## AUTHOR CONTRIBUTIONS

The author listed is the sole author.

## CONFLICT OF INTEREST

None to declare.

## CONSENT

This manuscript is a “Letter to Editor” considering the published case report by Kaomongkolgit and Sawangarun.^2^ There is no case of a patient in this “Letter to Editor” but only a hypothesis (risk factor).

## Data Availability

Data available on request from the author.

## References

[1] Troeltzsch M , Gogl M , Berndt R , Troeltzsch M . Oral lichen planus following the administration of vector‐based COVID‐19 vaccine (Ad26.COV2. S). Oral Dis. 2021. doi:10.1111/odi.14025. Epub ahead of print. PMC8661663 34543493

[2] Kaomongkolgit R , Sawangarun W . Oral lichen planus following mRNA COVID‐19 vaccination. Oral Dis. 2022. doi:10.1111/odi.14182. Epub ahead of print. PMC9115415 35263820

[3] Herzum A , Burlando M , Molle MF , Micalizzi C , Cozzani E , Parodi A . Lichen planus flare following COVID‐19 vaccination: a case report. Clin Case Rep. 2021;9(12):e05092. doi:10.1002/ccr3.5092. 34934493 PMC8650756

[4] Kulkarni R , Sollecito TP . COVID‐19 vaccination: possible short‐term exacerbations of oral mucosal diseases. Int J Dermatol. 2021;60(9):e335‐e336. doi:10.1111/ijd.15779. 34251026 PMC8444768

[5] Bergamaschi C , Terpos E , Rosati M , et al. Systemic IL‐15, IFN‐γ, and IP‐10/CXCL10 signature associated with effective immune response to SARS‐CoV‐2 in BNT162b2 mRNA vaccine recipients. Cell Rep. 2021;36(6):109504. doi:10.1016/j.celrep.2021.109504. 34352226 PMC8299183

[6] Gobbi F , Buonfrate D , Moro L , et al. Antibody response to the BNT162b2 mRNA COVID‐19 vaccine in subjects with prior SARS‐CoV‐2 infection. Viruses. 2021;13(3):422. doi:10.3390/v13030422. 33807957 PMC8001674

[7] Prendecki M , Clarke C , Brown J , et al. Effect of previous SARS‐CoV‐2 infection on humoral and T‐cell responses to single‐dose BNT162b2 vaccine. Lancet. 2021;397(10280):1178‐1181. doi:10.1016/S0140-6736(21)00502-X. 33640037 PMC7993933

